# Discovery and exploration of widespread infection of mycoviruses in *Phomopsis vexans*, the causal agent of phomopsis blight of eggplant in China

**DOI:** 10.3389/fpls.2022.996862

**Published:** 2022-11-10

**Authors:** Fang Ling Xie, Xin Yu Zhou, Rong Xiao, Chao Jun Zhang, Jie Zhong, Qian Zhou, Feng Liu, Hong Jian Zhu

**Affiliations:** ^1^ College of Plant Protection, Hunan Agricultural University, Changsha, China; ^2^ College of Horticulture, Hunan Agricultural University, Changsha, China; ^3^ Hunan Institute of Microbiology, Changsha, China

**Keywords:** phomopsis blight of eggplant, *Phomopsis vexans*, mycoviruses, mixed infections, deep sequencing

## Abstract

*Phomopsis vexans*, which causes Phomopsis blight of eggplant, has been reported worldwide. To study the biocontrol of this disease, 162 leaf and fruit samples of eggplant Phomopsis blight were collected from Hunan, Hubei, Jiangxi, Sichuan, Zhejiang, Fujian, Guangdong and Anhui Provinces from 2017 to 2019. Eighty-seven pathogenic fungus isolates were identified as *P. vexans.* The following studies were conducted: screening of sporulation medium, spore morphology analysis, mycovirus detection and identification of novel mycoviruses in these isolates. The results showed that eggplant tissue medium was the most suitable medium for rapid sporulation, and all isolates had mycoviruses consisting of mainly mixed infections. The genome of these mycoviruses varied from 1-15 kb. Five novel mycoviruses infecting *P. vexans* were obtained, including “*Phomopsis vexans* fusarivirus 1” (PvFV1), “*Phomopsis vexans* ourmia-like virus 1” (PvOLV1), “*Phomopsis vexans* endornavirus 2” (PvEV2), “*Phomopsis vexans* partitivirus 1” (PvPV1) and “*Phomopsis vexans* victorivirus L1” (PvVVL1). Thus, PvVVL1 displays a unique genome structure, and this is the first report of a victorivirus consisting of two segments and of a deltapartitivirus infecting the fungus host.

## 1 Introduction

Mycoviruses are viruses that infect and replicate in fungal cells (Hollings, 1962; Son et al., 2015). Most mycoviruses infect their fungal hosts asymptomatically (cryptic infections). However, several mycoviruses clearly can result in phenotypic alterations in the host, such as hypovirulence and debilitation. Therefore, mycoviruses have great potential to be exploited as biological agents against fungal diseases (Nuss, 2005; Jiang et al., 2013; Ghabrial et al., 2015; Li et al., 2019). Since *Cryphonectria hypovirus* 1 (CHV1) was successfully used to control chest blight disease in Europe (Nuss, 1992; Xie and Jiang, 2014; Ghabrial et al., 2015), an increasing number of hypoviruses have been detected in fungi, such as *Sclerotinia sclerotiorum*, *Botrytis cinerea*, and *Botryosphaeria dothidea* (Xiao et al., 2014; Hao et al., 2018; Azhar et al., 2019; Zhai et al., 2019). The mycovirus SSHADV-1 was used to transform the pathogenic fungus *Sclerotinia sclerotiorum* into an endophytic fungus that could coexist with, promote the growth and enhance the disease resistance of rape. The novel concept of a “plant vaccine” for the control of fungal diseases has been proposed, and it has also been pointed out that mycoviruses might be an important factor in the formation of endophytic fungi. Mycoviruses plays a significant role in the interaction between plants and fungi (Zhang et al., 2020). The development and application of high-throughput next-generation sequencing (NGS) technologies and bioinformatics have greatly enhanced the discovery of new viruses in many organisms, including fungi (Marzano et al., 2016; Zhang et al., 2018).

Some mycoviruses with attenuated virulence may not affect the growth rate of the host after infection but show physiological phenotypic changes under abiotic stress. For example*, Penicillium digitatum* polymycovirus 1 (PdPmV1) and *Penicillium digitatum* Narna-like virus 1 (PdNLV1) have no significant effect on the growth morphology of the host when coinfected with *Penicillium digitatum*; however, they reduce the sensitivity of the host to fungicides (Niu et al., 2018). Torres-Trenas et al., using a GFP fluorescence labeling technique, demonstrated that mycoviruses in *Fusarium oxysporum* affect its fixed speed and spatial distribution in plant roots (Torres-Trenas et al., 2019).

Mycoviruses can also promote the virulence of their fungal hosts. Curvularia thermal tolerance virus (CThTV) in the endophytic fungus *Curvularia protuberate* can enhance the heat tolerance of its fungal host and the plants simultaneously (Marquez et al., 2007), which provides material for studying the tripartite interaction among viruses, fungi and plants. Kotta-Loizou and Coutts found that mycoviruses can improve the pathogenicity of *Beauveria bassiana* to *Galleria mellonella* (Kotta-Loizo and Coutts, 2017). *Talaromyces marneffe* partitivirus 1 (TmPV1) can enhance the pathogenicity and tolerance of host fungi and increase the lethality of the host to mice in *Talaromyces marneffe* (Lau et al., 2018).

Mycoviruses are divided into five categories according to the nucleic acid type: retroviruses, negative single-stranded RNA viruses, positive single-stranded RNA viruses, double-stranded RNA viruses and DNA viruses. Double-stranded RNA viruses are divided into 7 families: Totiviridae, Partitiviridae, Chysoviridae, Endornaviride, Reoviridae, Quadriviridae and Megabirnaviridae. Negative single-stranded RNA viruses include the single-stranded negative-stranded RNA virological family (Mymonaviridae); DNA viruses comprise the Geminivirus-like family (Genomoviridae); retroviruses include the transposable virofamily (Metaviridae) and pseudoviridae (Pseudoviridae); positive single-stranded RNA viruses approved by the International Commission for the Classification of Virology include eight families: Alphaflexiviridae, Gammaflexiviridae, Deltaflexiviridae, Barnaviridae, Botourmiaviridae, Narnaviridae, Hypoviridae and Endornaviridae (King et al., 2012). Fusariviridae is a recently proposed family of + ssRNA viroviridae. The genome size of Fusariviruses is 6-10 kbp, and the larger ORF encodes replicase-related proteins.

In recent decades, a number of phytopathogenic and plant-associated fungi have been screened for viruses from a few perspectives, such as virological control practices (biological use of viruses) and basic virus research. The tested fungi were shown to be infected by viruses at different incidence rates, ranging from a few to over 90% (Hillman et al., 2018). These studies have revealed the great diversity of fungal viruses and provided insights into their evolutionary histories (Xie and Jiang, 2014; Marzano and Domier, 2016; Marzano et al., 2016). For example, dsRNA analysis has revealed that of the 79 *R*. *necatrix* isolates tested (62 Spanish, 1 Italian and 16 Israeli), 11 tested positive for the presence of dsRNA (Arjona-López et al., 2018). Luo isolated 13 *B. dothidiea* strains in Wuhan city of Hubei Province. After dsRNA detection, 12 were found to carry mycoviruses, and the carrying rate was as high as 92.31% (Luo et al., 2019). All 31 *Entoleuca* sp. isolates showed mycovirus infection. New virus-carrying Entoleuca sp. isolates were obtained, which are capable of controlling avocado white root rot (Arjona-López and López-Herrera, 2021). All *P. vexans* isolated from eggplant in this study also carried mycoviruses.

Eggplant is an economically important crop with a long cultivation history. It is widely planted in China, India, Pakistan, Menggara, the Philippines and other countries (Kumar et al., 2011). Eggplant brown streak (Phomopsis blight of eggplant) caused by *P. vexans* is an important disease that limits eggplant production (Rangaswami and Madevan, 1998; Khan, 1999; Akhtar and Chaube, 2006; Jayaramaiah et al., 2013), causing leaf and stem death or fruit rot. The disease was shown to decrease the production of eggplant by 20-50% in one season (Akhtar and Chaube, 2006; Pandey, 2010). The pathogen of eggplant brown streak can pass through the infected seed coat, cotyledon and hypocotyl (Thippeswamy et al., 2012), resulting in a low seed germination rate and poor seed vigor. The seed yield is often reduced or even lost in breeding fields due to the spread of the disease (Das, 1998; Beura et al., 2008; Udayashankar et al., 2019). Eggplant brown streak was first reported in Nanjing, China in 1932 (Teng, 1932). At present, the disease has spread to most parts of the world (Smith et al., 1988) (Supplementary Figure SEQ Figure \* ARABIC 1). In addition to infection of eggplant, Phomopsis comprises more than 100 different species and can live in more than 70 different families of herbaceous and woody plants and cause serious disease in soybean and sunflower oil crops, such as vegetables and fruit trees. These disease include soybean satisfactory stem rot (Jackson et al., 2009), Phomopsis stem cStem anker of sunflower Sunflower (Hulke et al., 2019), asparagus stem blight (Zhang et al., 2012), and die-back disease of neem (Nagendra Prasad et al., 2010), a very important plant pathogenic fungus. Mycoviruses reported in *P. vexans* include only *P. vexans* RNA virus 1 (PvRV1) (Zhang et al., 2015) and *P. vexans* partitivirus 1 (PvPV1) (Zhang et al., 2021). In this study, a number of *P. vexans* strains were isolated 58 eggplant planting areas in eight provinces of China.All *P. vexans* isolated in this study carried mycoviruses, which contained not only rich mycovirus resources but also the possibility of mycoviruses that could be used as plant vaccines.The genome size of the mycovirusesvaried from 1 to 15 kbp, and most of the strains showed mixed infection. Eggplant brown stripe pathogens are rich in mycoviruses resources.

## 2 Materials and methods

### 2.1 Fungal strains

The *P. vexans* strains were originally isolated from a typical disease lesion collected from eggplants in China. The isolates were maintained at 25°C on potato dextrose agar and stored at 4°C in the dark. Mycelia were cultured in potato dextrose broth (potato, glucose) liquid medium in an orbital shaker at 25°C for 5 to 7 days for nucleic acid extraction.

### 2.2 Observation of the spore carrier and spore morphology of *P. vexans* strains

The main basis for identification of the fungi of *P. vexans* strains is to observe the morphology of the two conidia. We attempted to use PDA medium (200 g potato, 20 g glucose, 15 g agar, followed by filtering with gauze and setting the volume to 1000 ml), OMA medium (20 g oat boiled for 1 h, followed by filtering with gauze and setting the volume to 1000 ml) and eggplant tissue culture medium to promote the sporulation of *P. vexans* strains. A large amount of sporulation could be observed on the decomposed eggplant fruit in the field. Fresh mycelial blocks were inoculated on PDA, OMA and eggplant tissue culture media and cultured at 25°C in the dark.

### 2.3 dsRNA extraction and purification

Mycelia of *P. vexans* strains were cultured in potato dextrose broth at 25°C for 7 days. The mycelia were collected and then finely ground using liquid nitrogen. The cellulose CF-11 method was used to extract the dsRNA. DNA and ssRNA contaminants were eliminated from the dsRNA by digestion with DNase I and S1 nuclease (Thermo). The dsRNA in each extract was dissolved in diethypyrocarbonate-treated water and then visualized by electrophoresis on a 1% (w/v) agarose gel stained with nucleic acid dye. The dsRNA segments extracted from *P. vexans* were purified using a Mini BEST Agarose Gel DNA Extraction Kit.

### 2.4 cDNA cloning, sequencing, and phylogenetic analysis

Conventional cDNA library construction was performed for viral sequence determination. After denaturation of dsRNA templates at 65°C for 5 min, cDNA was synthesized using Revert Aid M-MuLV with an adapter-tagged primer (5’-CGATCGATCATGATGCAATGCNNNNNN-3’) and amplified with a specific primer (5’- CGATCGATCATGATGCAATGC-3’). To obtain the terminal sequences of mycoviruses, the 5’-end phosphorylated oligonucleotide AdaptorA 5’-TCTCTTCGTGGGCTCTTGCG-(NH2)-3’ was used for ligation of dsRNA using T4 RNA ligase at 16 °C for 16 h and was then reverse transcribed using primer B (5’-CGCAAGAGCCC ACGAAGAGA- 3’). The cDNA terminal strands were then used as templates for PCR amplification of the 5’- and 3’- terminal sequences with primer B. All of the amplified DNA fragments were cloned into the pMD18-T vector or pClone007-Blunt cloning vectors and used for the transformation of *Escherichia coli* strain DH51α.

After removing the coupling primer and vector sequences, DNAMAN software was used for splicing and sequencing according to the virus fragments. Primer 5.0 was used to design the primers. The NCBI conserved domain database was used to perform a conservative structure domain search (http://www.ncbi.nlm.nih.gov/Structure/cdd/wrpsb.cgi). The ORFs of the viruses were predicted based on standard genetic codes, and their homologous aa sequences were searched in the NCBI database using ORF finder and the BLASTp programs. Sequence alignment was performed using CLUSTALX. Annotation of the conserved sequences was carried out using GeneDoc. A phylogenetic tree was constructed using MEGA software version 7.0 by the neighbor-joining (NJ) method, with 1000 bootstrap replicates.

## 3 Results

### 3.1 Isolation and purification of *P. vexans* strains

**Figure 1:**
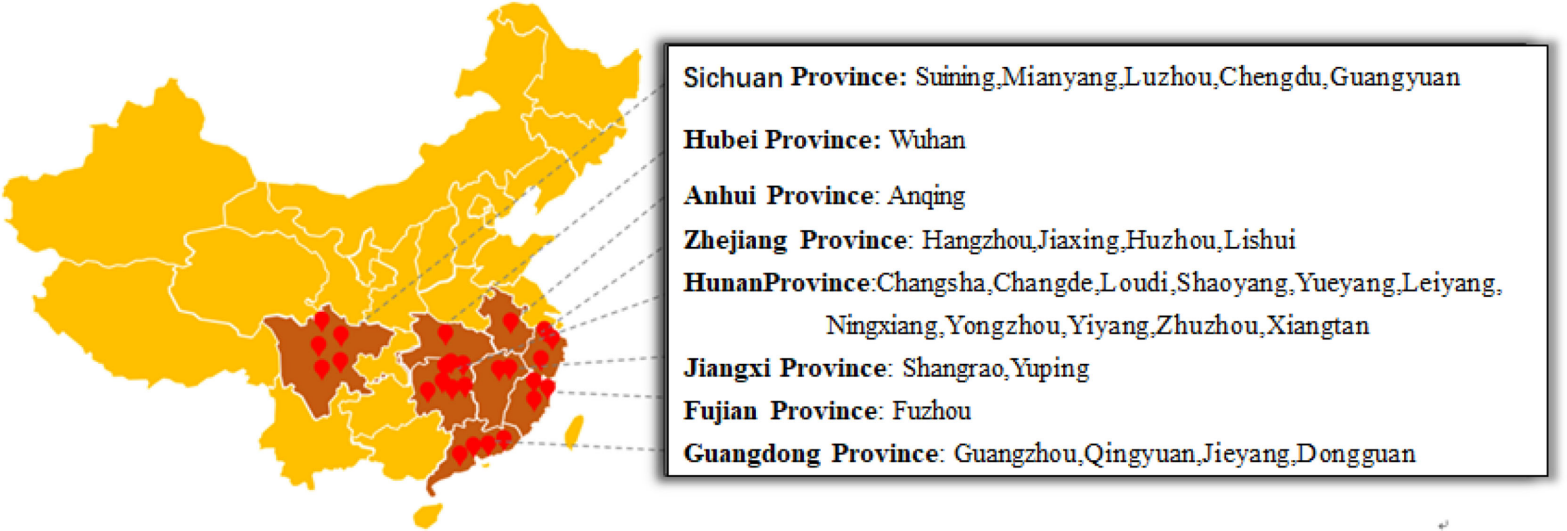
Sample collections of Phomopsis blight of eggplant and distribution in China.

**Figure 2:**
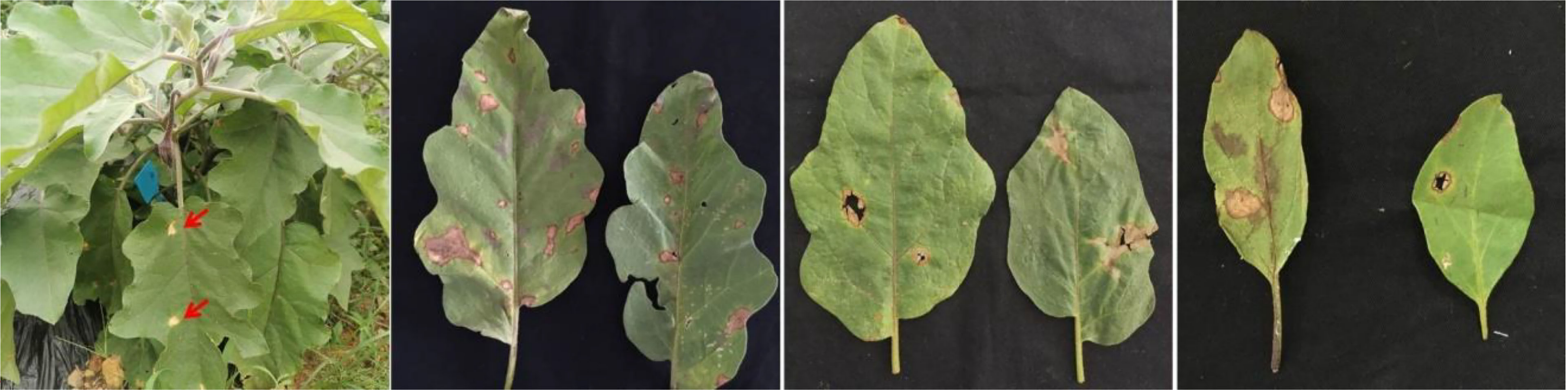
Field symptoms of leaf blight of *P. vexans*.

From 2017 to 2019, 162 leaf and fruit samples with typical symptoms of eggplant brown streak were collected from 58 eggplant planting areas in Hunan, Hubei, Jiangxi, Sichuan, Zhejiang, Fujian, Guangdong and Anhui Provinces. The location and geographical distribution of the samples in the field are shown in **Figure 1**, and the symptoms of eggplant brown streak are shown in **Figure 2**.

**Figure 3:**
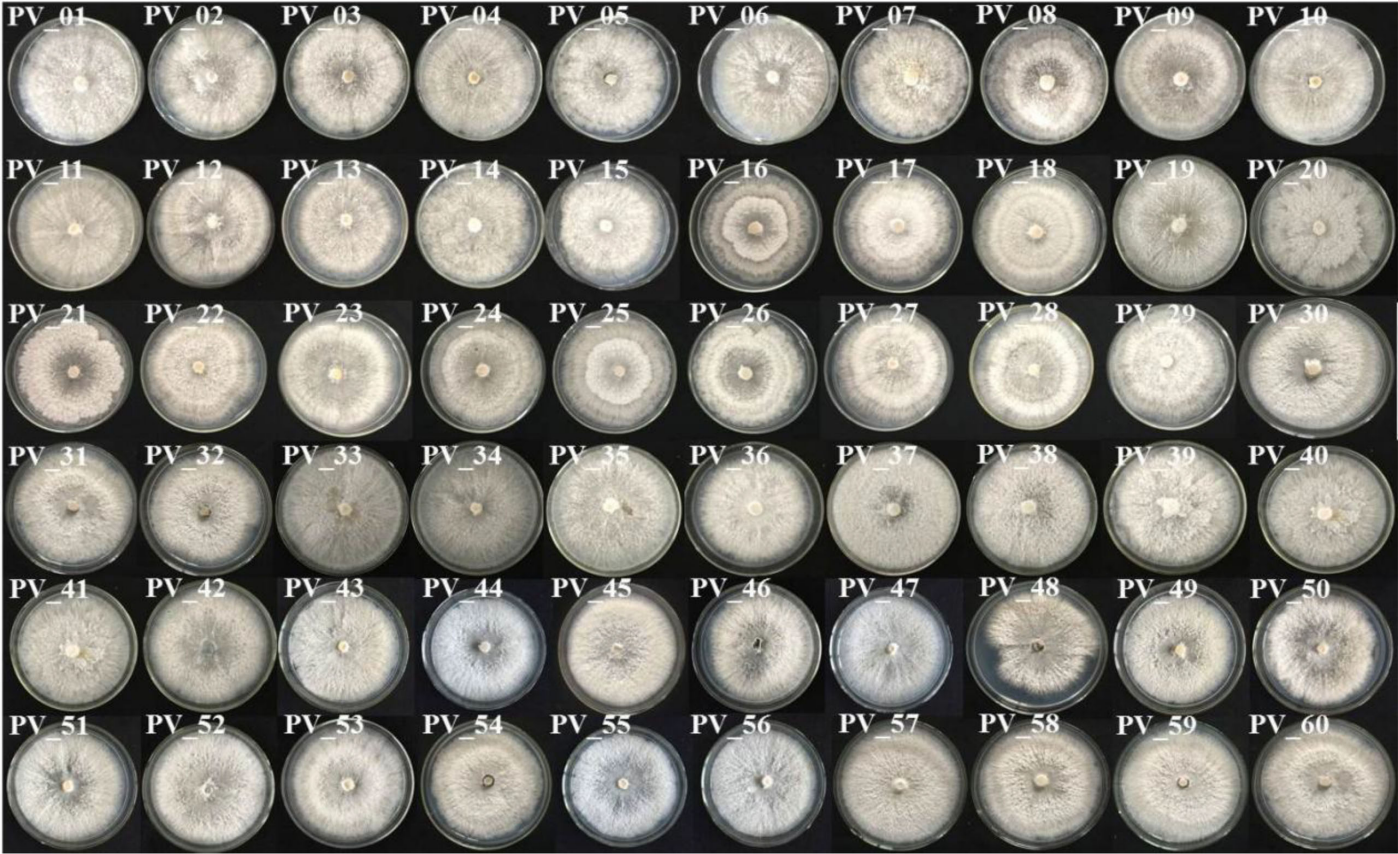
Colony morphology of *P. vexans* isolate strains on potato dextrose agar medium after 7 days of incubation at 25°C.

A total of 87 P*. vexans* strains were isolated, which included some Colletotrichum and Alternaria, but none of them were our target strains. *P. vexans* colonies were first white and then became yellowish, ultimately resulting in the formation of black pycnidium. By observing the colony morphology, colonies typical of *P. vexans* were selected for purification. The morphology of the isolated *P. vexans* strains is shown in **Figure 3**.

### 3.2 Observation of the spore carrier and spore morphology of *P. vexans* strains

**Figure 4:**
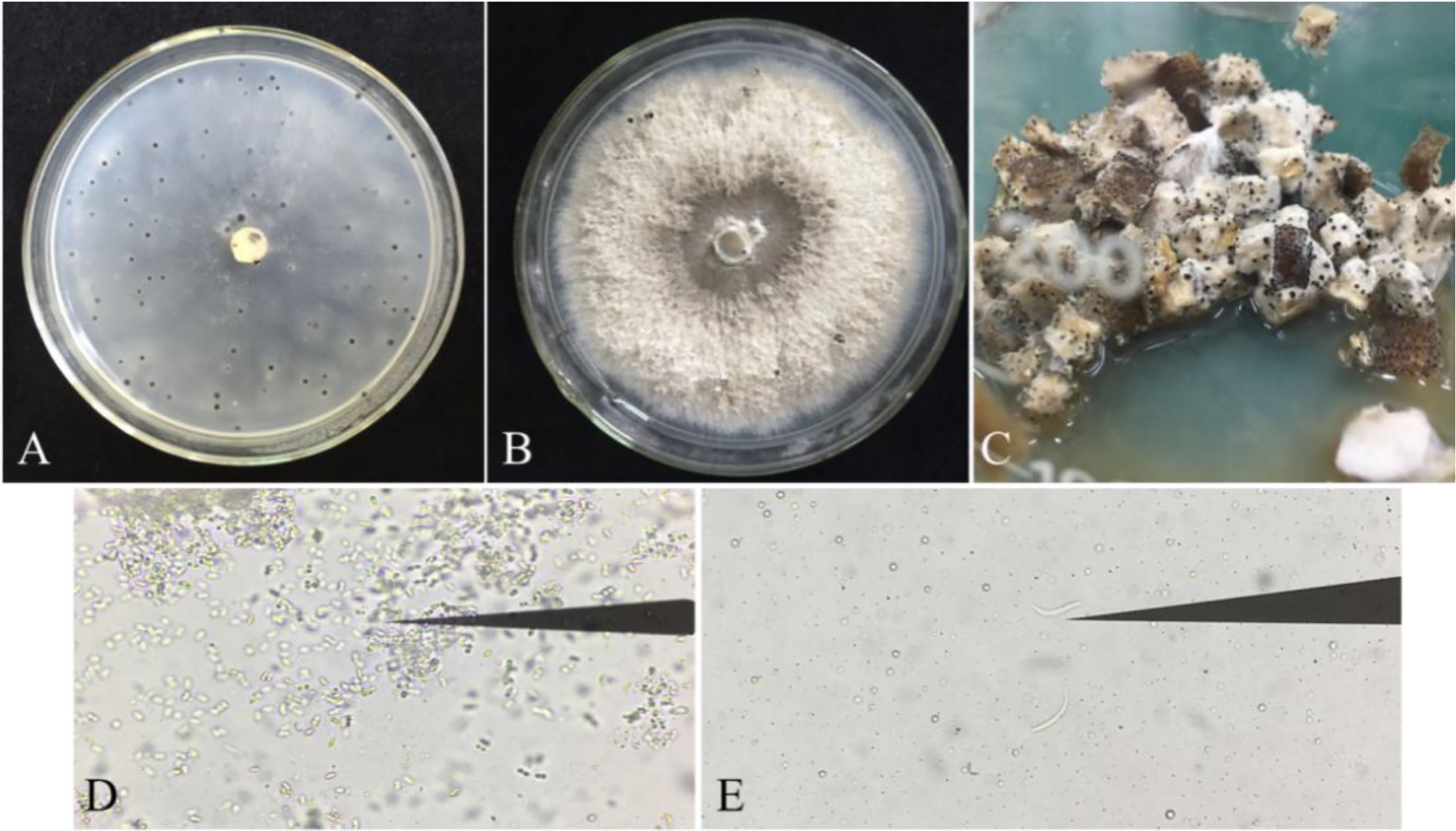
Pycnidia of *P. vexans* on different media. **(A)** Colony morphology of *P. vexans* Pycnidia on oatmeal agar medium after 30 days of incubation at 25°C. **(B)** Colony morphology of *P. vexans* Pycnidia on potato dextrose agar medium after 30 days of incubation at 25°C. **(C)** Colony morphology of *P. vexans* Pycnidia on *S. melongena* fruit medium after 5 days of incubation at 25°C. **(D)** α-type conidia under a compound microscope (400 X). **(E)** β-type conidia under a compound microscope (400 X).

The purified strains were identified with ITS4/ITS5 primers. Sequence homology comparison identified 87 strains as *P. vexans*. The pycnidium of *P. vexans* was induced and cultured on three kinds of media. The results showed that the number of pycnidium was greater on OMA medium than on PDA and eggplant decoction medium in the dark at 25 °C. However, the eggplant tissue culture medium was most suitable for spore production; many pycnidia emerged after 5-7 days of cultivation, and the sporulation load of a single pycnidium was the largest (**Figures 4A–C**). The α-type and β-type conidia of *P. vexans* were observed in the pycnidium (**Figures 4D, E**), which was consistent with the morphological characteristics of *P. vexans*.

### 3.3* P. vexans* contain abundant dsRNAs

**Figure 5:**
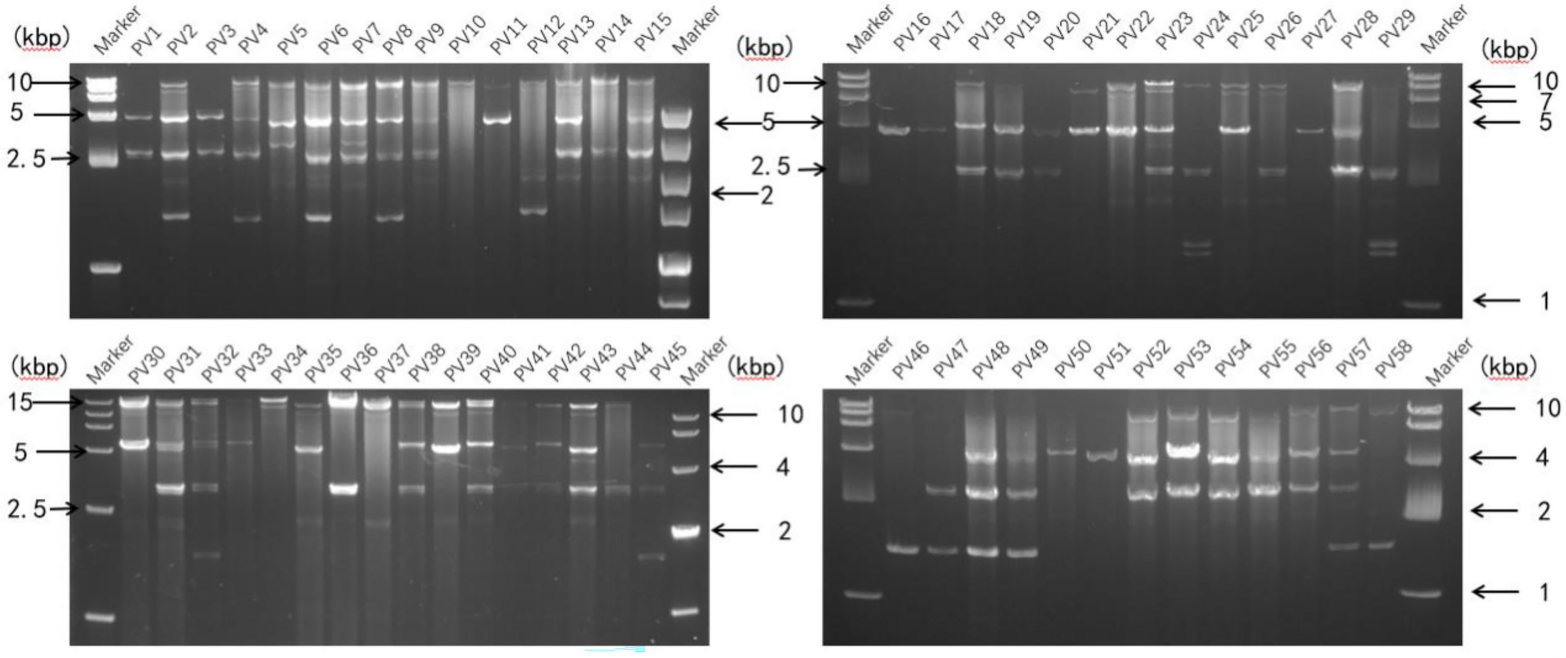
Agarose gel electrophoresis analysis of dsRNA extracted from *P. vexans.* Chromatography of cellulose extracts from the *P. vexans* strain showing dsRNA bands indicative of mycovirus infection.

A representative *P. vexans* strain was selected from each eggplant planting area, making up all 58 strains, to detect dsRNA viruses (**Supplementary Table 1**). Nucleic acid extraction resulted in a number of nucleic acid bands (**Figure 5**) that were resistant to DNase I and S1 nuclease digestion, indicating that the presence of dsRNA-like nucleotides was 100%, and the results showed abundant mycoviral resources in the *P. vexans* strain. The 58 isolated strains all had detectable mycoviruses. The genome size of the mycoviruses varied from 1 to 15 kbp, and most of the strains showed mixed infection. Only 6 strains were infected with a single dsRNA virus (PV_05, PV_06, PV_14, PV_15, PV_25, PV_37), accounting for 10.3% of the tested strains. According to the statistical analysis of dsRNA bands of different sizes, 77.6% of the tested strains were infected with nucleic acid bands of approximately 5.0 kb in size (45/58), demonstrating the highest carrying rate. Nucleic acid bands greater than 10 kb in size (43/58) accounted for 74.1% of the tested strains. There were differences in the detection rate of dsRNA among strains isolated from different provinces. The top three average carrying rates of dsRNA were from Anhui, Sichuan and Hunan, while the lowest was isolated from Guangdong (the number of strains from Jiangxi and Hubei was less than 3, which was not biologically significant and thus was not taken into account (**Table 1**). Nucleic acid bands of approximately 3.0 kb in size were usually accompanied by other mycoviruses.

**Table 1:** Table of dsRNA bands in *P. vexans*.

Provinces of strain isolation	Number of strains	Toal number of dsRNA	The average number of dsRNA
Anhui	3	21	7
Sichuan	7	33	4.7
Hunan	27	118	4.37
Zhejiang	6	23	3.83
Fujian	7	25	3.57
Guangdong	5	6	1.2
Jiangxi	2	7	–
Hubei	1	4	–

### 3.4 Identification of novel mycoviruses

High-throughput sequencing was carried out using a mixture of 10 strains in 6 batches. In this study, we identified diverse viral segments from *P. vexans* strains using high-throughput transcriptome sequencing. Some viruses discovered in our analysis were nearly full length and included 5 unreported viruses. 5 whole viral genomes were obtained by random primer construction library and ligase-mediated end amplification. Their complete nucleotide sequences were assembled by DNAMAN. The predicted amino acid sequences of the putative viral genomes showed significant sequence identity with described viruses from several distinct lineages, including one novel virus in the family Botourmiaviridae, named *P. vexans* ourmia-like virus 1 (PvOLV1), one novel virus in the family Fusariviridae, named *P. vexans* fusarivirus 1 (PvFV1), one novel virus in the family Endornaviridae, named *P. vexans* endornavirus 2 (PvEV2), one novel virus in the family Partitiviridae, named *P. vexans* partitivirus 1 (PvPV1) and one novel virus in the family Totiviridae in which an unconventional victorivirus consisted of two segments of nucleic acid, named *P. vexans* lexivirus L1 (PvVVL1).

#### 3.4.1 Analysis of the PvOLV1 genome structure

**Figure 6:**
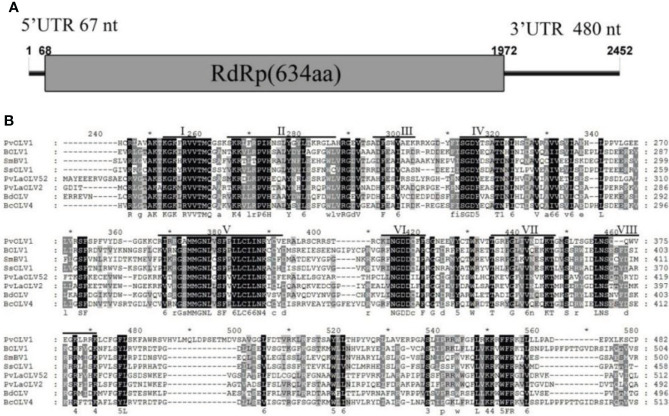
Diagrammatic representation of the genome organization and multiple alignment of PvOLV1. **(A)** Schematic representation of the PvOLV1 genome organization, containing a single open reading frame (ORF) encoding a putative RNA-dependent RNA polymerase (RdRp) and 5’ and 3’ untranslated regions (UTRs). **(B)** Multiple alignment of the aa sequences of RdRp domains of PvOLV1 and other similar Botourmiaviridae viruses using the ClustalX program and highlighted using the GeneDoc program. The conserved motifs in these RdRps are indicated by Roman numerals I to VIII. Virus names and accession numbers are as follows: BOLV1, Botrytis ourmia-like virus (QLF49182.1); SmBV1, *Sclerotinia minor* botoulivirus 1 (QHR78948.1); SsOLV1, *Sclerotinia sclerotiorum* ourmia-like virus 1 (ALD89138.1); PvLaOLV52, *Plasmopara viticola* lesion associated ourmia-like virus 52 (QGY72582.1); PvLaOLV2, *Plasmopara viticola* lesion associated ourmia-like virus 2 (QGY72532.1); BdOLV, *Botryosphaeria dothidea* ourmia-like virus (QED22728.1); BcOLV4, *Botrytis cinerea* ourmia-like virus 4 (QJT73670.1).

Sequence analysis of the full-length cDNA indicated that the PvOLV1 genome (**Figure 6A**) was 2452 nt in length and had a C +G content of 54.73%. ORF prediction revealed that PvOLV1 consisted of a unique ORF of 1904 nt (nt positions 68–1972), which encoded a 634-amino-acid (aa)-residue polypeptide with a calculated molecular mass of 72.04 kDa. The 5’-untranslated region (UTR) of PvOLV1 was determined to be 67 nt long. The 3’-UTR was relatively long, at 480 nt. BLASTp alignment of its RdRp indicated that PvOLV1 showed the highest aa identity (E-value: 3e-110; query cover: 95%; identity: 38.7%) with *Plasmopara viticola*-associated ourmia-like virus 52 (PvLaOLV52). A search of the conserved domain database (CDD) and multiple protein alignment confirmed that the predicted RdRp domains contained eight conserved motifs (I to VIII), including the GDD motif, a typical characteristic of mycoviral RdRps (**Figure 6B**). We have named this novel mycovirus, the first reported in this fungal pathogen, *P. vexans* ourmia-like virus 1 (PvOLV1). The virus sequence has been deposited in GenBank under accession number MZ044287.

**Figure 7:**
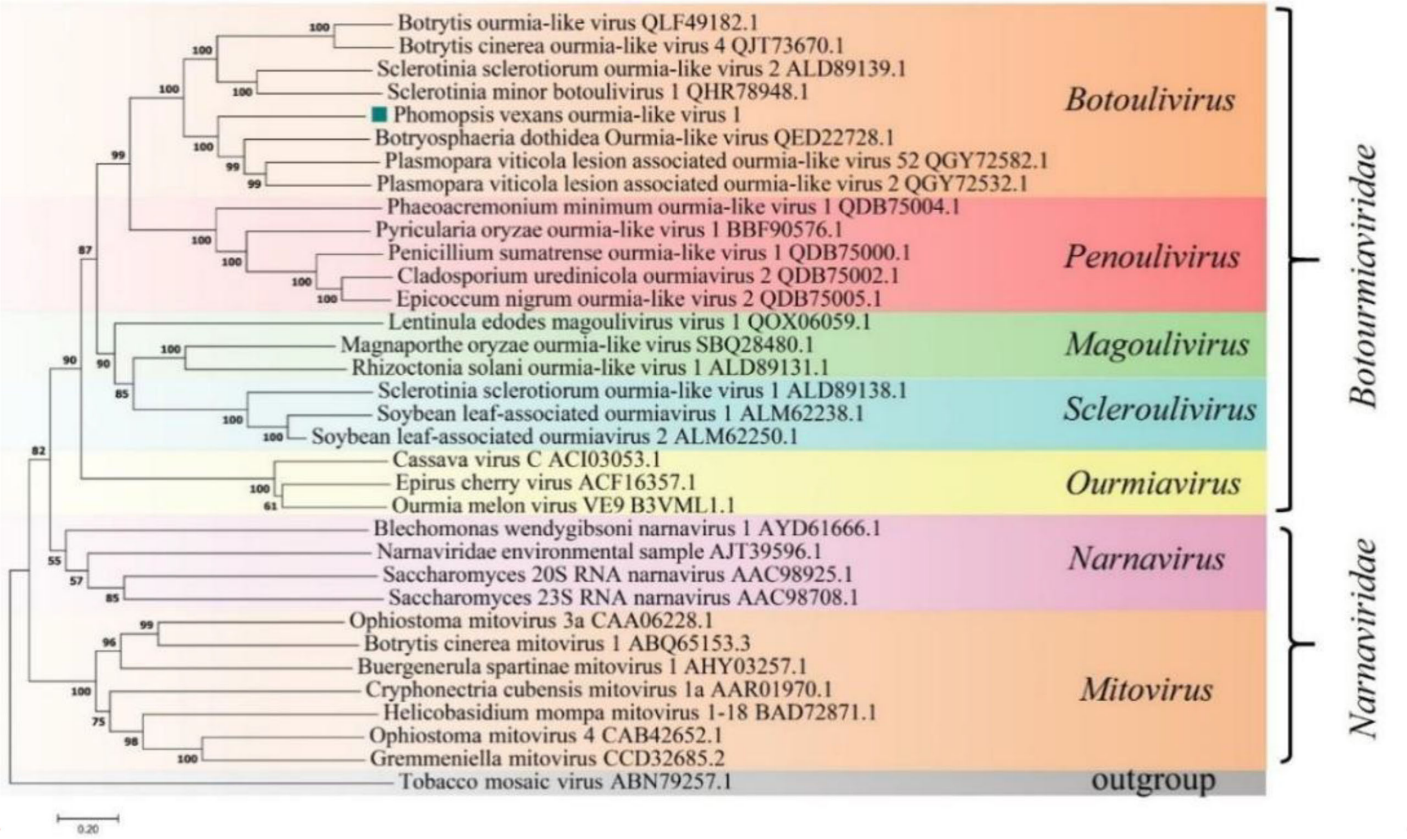
Phylogenetic analysis of PvOLV1. Phylogenetic analysis of PvOLV1 based on aa alignments of RdRp and other viruses related to these proteins. A phylogenetic tree was constructed by the neighbor-joining algorithm using MEGA7, with 1000 bootstrap replications. The percentage of bootstrap values supporting the branches in phylogenetic trees are indicated on the nodes. The genetic distance is represented by the scale bar of 0.2 aa substitutions per site. The novel virus PvOLV1 is indicated by a green square. The names and database accession numbers of other related viruses analyzed are indicated in the tree.

A phylogenetic tree was constructed based on the deduced aa sequence of the putative RdRp region encoded by PvOLV1 using the NJ method in MEGA (version 7.0). The results showed that PvOLV1 was clustered with ourmiavirus in the family Botourmiaviridae (**Figure 7**).

#### 3.4.2 Analysis of the genomic structure of PvFV1

**Figure 8:**
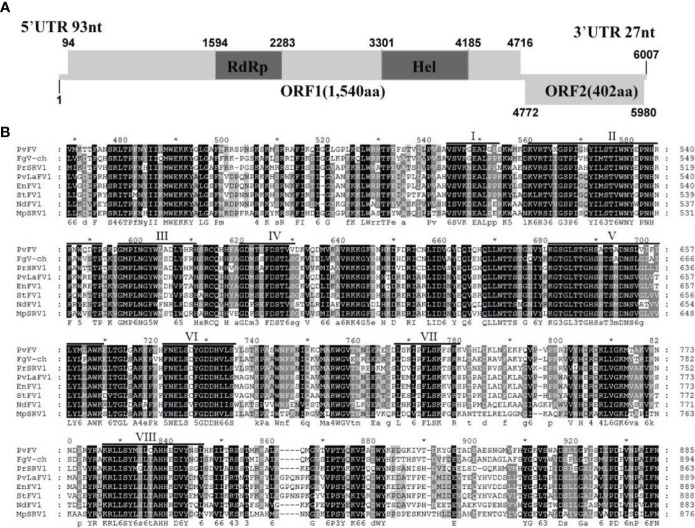
Diagrammatic representation of the genome organization and multiple alignment of PvFV1. **(A)** Schematic representation of the PvFV1 genome organization, which contained a single open reading frame (ORF) encoding a putative RNA-dependent RNA polymerase (RdRp) and 5’ and 3’ untranslated regions (UTRs). **(B)** Multiple alignment of the aa sequences of the RdRp domains of PvFV1 and other similar Fusariviruses using the ClustalX program and highlighted using the GeneDoc program. The conserved motifs in these RdRps are indicated by Roman numerals I to VIII. Virus names and accession numbers are as follows: FgV-ch, *Fusarium graminearum* dsRNA mycovirus-1 (AAT07067.2); PrSRV1, *Penicillium roqueforti* ssRNA mycovirus 1 (AII99895.1); PvLaFV1, *Plasmopara viticola* lesion associated fusarivirus 1 (QHD64725.1); EnFV1, *Erysiphe necator*-associated fusarivirus 1 (QHD64833.1); StFV1, *Setosphaeria turcica* fusarivirus 1 (QRI93681.1); NdFV1, *Neurospora discreta* fusarivirus 1 (BCL64192.1); MpSRV1, *Macrophomina phaseolina* single-stranded RNA virus 1 (ALD89094.1).

Sequence analysis of the full-length cDNA indicated that the PvFV1 genome (**Figure 8A**) was 6007 nt in length, excluding the poly(A) structure at the end of the 3’ noncoding region, and had a C+G content of 42.86%. The 5’ and 3 ‘noncoding regions were 93 nt and 27 nt long, respectively. Fusariviridae is a new, recently proposed + ssRNA family comprising members that typically have 6 to 8 kbp genomes, with one larger ORF encoding putative polyproteins of replicases and one to three smaller ORFs encoding hypothetical proteins (Zhang et al., 2014). The genome of PvFV1 also contained two ORFs that did not overlap. ORF1 encoded RdRp, which was predicted to encode a protein of 1540 aa with a molecular weight of 174.75 kDa. ORF2 was predicted to encode a 402-aa protein with a molecular weight of 45.78 kDa. CD-Search in NCBI indicated that ORF1 contained a conserved RdRp and an RNA unwinding domain, and ORF2 contained a DUF3084 (e-value, 4.31e-04) domain with unknown function. BLASTp alignment of its ORF1 revealed the highest aa identity (E-value: 0; query cover: 94%; identity: 51.7%) with the RdRp of the Fusariviridae family, *Fusarium graminearum* dsRNA mycovirus-1 (FgV-ch). CD search showed that the ORFs contained eight conserved domains of the Fusariviridae family virus (**Figure 8B**). The virus sequence was deposited in GenBank under accession number MZ044286.

**Figure 9:**
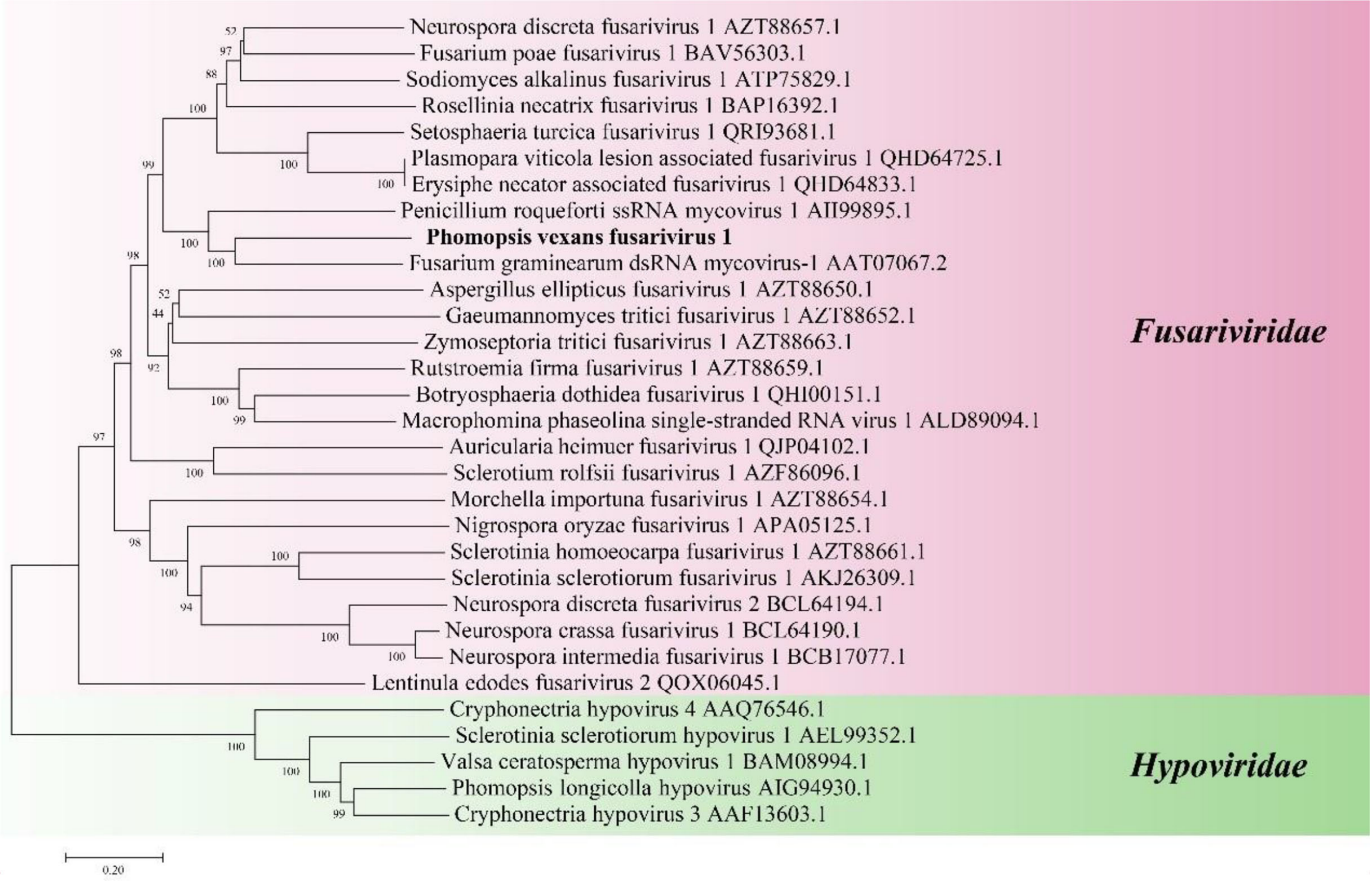
Phylogenetic analysis of PvFV1. Phylogenetic analysis illustrating the evolutionary classification of PvFV1 was conducted using the method described in **Figure 7**. The scale bar represents a genetic distance of 0.2 aa substitutions per site. The black bold font indicates the novel mycoviruses PvFV1 identified in the present study. The names and database accession numbers of other related viruses analyzed are indicated in the tree.

The phylogenetic tree of PvFV1 encoding RdRp aa was constructed using MEGA7 software, the NJ method and the Poisson model. The results showed that PvFV1 and Fusariviridae were clustered together (**Figure 9**).

#### 3.4.3 Genomic structure analysis of PvEV2

**Figure 10:**
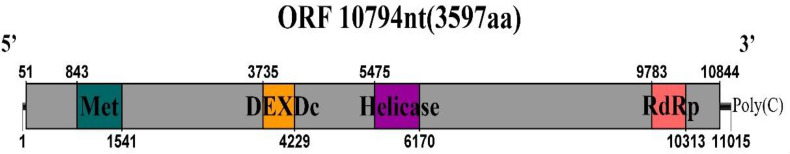
Diagrammatic representations of the predicted genomic organization of PvEV2. Comparisons of the genomic organizations of the novel *P. vexans* infecting PvEV2 and the identified member PvEV2 of the Endornaviridae family. PvEV2 was completely sequenced and predicted to harbor a single larger ORF containing domains of viral methyltransferase (MTR), putative DEXDc, viral helicase (Hel) and RdRp, which are indicated in the ORF box.

The sequences showed similarity to members of the family Endornaviridae. Viruses in the Endornaviridae family have linear ssRNA genomes ranging in length from approximately 10 kb to more than 17 kb and containing an ORF encoding a single long polyprotein. The polyprotein encoded by endornaviruses often includes conserved domains, such as viral RNA helicases and RdRps (Ghabrial et al., 2015). We designated the virus *P. vexans* endornavirus 2 (PvEV2). The full-length PvEV2 genome was 11015 nt, excluding the poly(C) structure at the end of the 3’ noncoding region, which contained a single ORF encoding a 3597-aa polyprotein with a molecular weight of 397.10 kDa (**Figure 10**). The BLASTP alignment information of the four conserved domains of PvEV2 with other Endornaviruses are shown in **Table 2**. Domain searches revealed that this polyprotein contained the conserved domains of viral methyltransferase (Met), putative DEXDc, viral helicase (Hel) and RdRp. Consistent with the homology search, phylogenetic analysis based on the conserved RdRp domain suggested that PvEV2 was a new putative species in the family Endornaviridae. The virus sequence was deposited in GenBank under accession number MZ044288.

**Table 2:** Percent aa sequence identity of the 2 conserved domains of *P. vexans* endornavirus compared with those of other endornaviruses.

Protein	Organism name	Accession number	Identify	e-value
RdRp	*Botrytis cinerea* betaendornavirus 1	YP_009315910.1	73.86%	4E-86
	*Sclerotinia sclerotiorum* endornavirus 2-A	AWY10955.1	74.43%	2E-85
	*Gremmeniella abietina* type B RNA virus XL1	YP_529670.1	75.57%	6e-85
	*Gremmeniella abietina* type B RNA virus XL2	ABD73306.1	75%	7E-85
	*Sclerotinia sclerotiorum* endornavirus 2	AND83000.1	73.86%	2e-84
Hel	*Gremmeniella abietina* type B RNA virus XL1	YP_529670.1	36.36%	1E-37
	*Gremmeniella abietina* type B RNA virus XL2	ABD73306.1	35.93%	2E-37
	*Sclerotinia sclerotiorum* endornavirus 2	AND83000.1	33.91%	4E-35
	*Sclerotinia sclerotiorum* endornavirus 1	AJF94392.1	33.48%	1E-34
	*Sclerotinia sclerotiorum* betaendornavirus 1	YP_009022070.1	33.91%	3E-34
Met	*Sclerotinia sclerotiorum* endornavirus 2	AND83000.1	54.31%	1E-78
	*Sclerotinia sclerotiorum* betaendornavirus 1	YP_009022070.1	53.45%	2E-77
	*Botrytis cinerea* betaendornavirus 1	YP_009315910.1	53.88%	2E-76
	*Sclerotinia sclerotiorum* endornavirus 1	AJF94392.1	53.02%	3E-76
	*Gremmeniella abietina* type B RNA virus XL2	ABD73306.1	49.79%	7E-76
DEXDc	*Gremmeniella abietina* type B RNA virus XL2	ABD73306.1	44.79%	4E-32
	*Gremmeniella abietina* type B RNA virus XL1	YP_529670.1	44.79%	4E-32
	*Sclerotinia sclerotiorum* endornavirus 1	AJF94392.1	40.65%	4E-32
	*Sclerotinia sclerotiorum* endornavirus 1	YP_008169851.1	41.29%	5E-32
	*Sclerotinia sclerotiorum* endornavirus 1	YP_009022070.1	38.71%	1E-29
	*Sclerotinia sclerotiorum* endornavirus 2	AND83000.1	38.06%	1E-28

**Figure 11:**
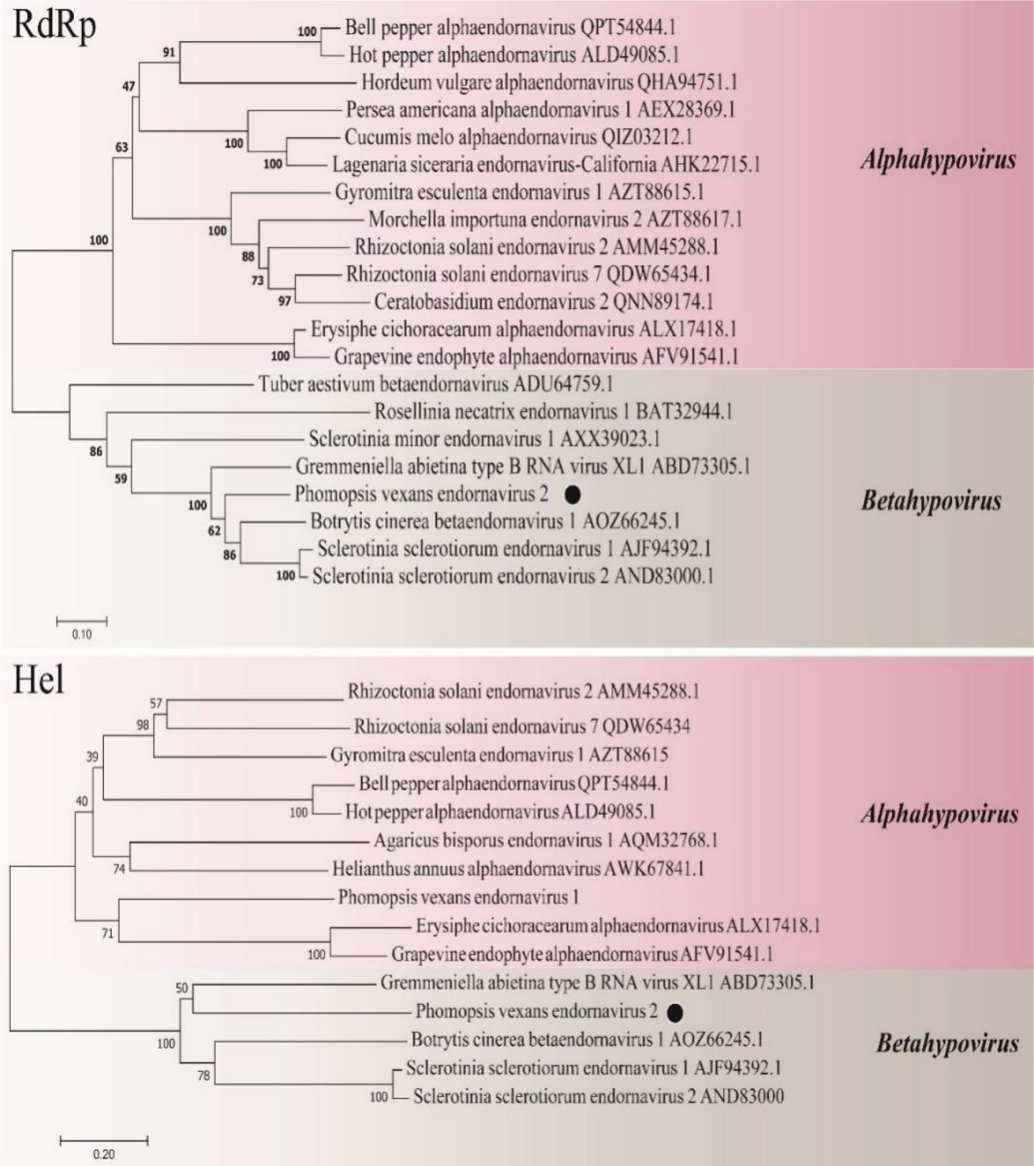
Phylogenetic analysis of PvEV2. Phylogenetic analysis illustrating the evolutionary classification of PvEV2 was conducted using the method described in **Figure 7**. The genetic distance is represented by the scale bar of 0.2 aa substitutions per site. The novel virus PvEV2 is indicated by black dots. The names and database accession numbers of other related viruses analyzed are indicated in the tree.

A phylogenetic tree was constructed using the neighbor-joining algorithm in MEGA7, with 1000 bootstrap replications (**Figure 11**). PvEV2 and Betaendornavirus in the family Endornaviridae were clustered together. There was a poly(C) structure at the end of the 3’ noncoding region of PvEV2 and a methyltransferase domain rather than a glycosyltransferase domain. It was inferred that PvEV2 was a betaendornavirus.

#### 3.4.4 Structural analysis of the PvPV1 genome

**Figure 12:**
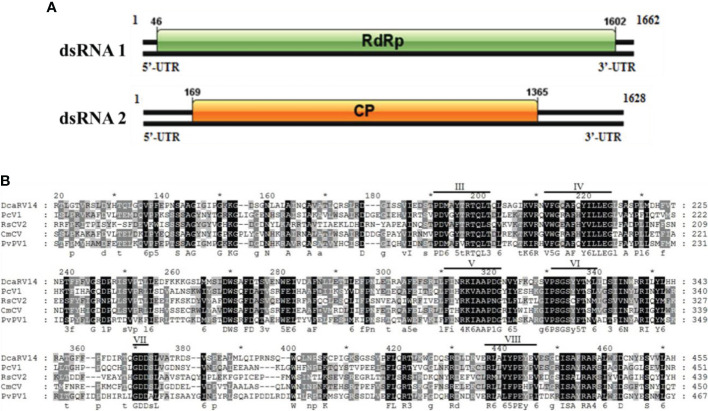
Diagrammatic representation of the genome organization of PvPV1. **(A)** Diagram showing the structure of PvPV1. The open reading frame (ORF) and the 5’ and 3’ untranslated regions (UTRs) are indicated by the colored long rectangular box and solid black lines, respectively. The initiation and termination codons of the ORF are indicated by the numbers under the solid lines. **(B)** Multiple alignment of the aa sequences of RdRp domains of PvPV1 and other similar deltapartitiviruses using the ClustalX program and highlighted using the GeneDoc program. The conserved motifs in these RdRps are indicated by Roman numerals III to VIII. Virus names and accession numbers are as follows: DcaRV14, diatom colony-associated dsRNA virus 14 (AP014906); PcV1, pepper cryptic virus 1 (AVV48358); RsCV2, *Raphanus sativus* cryptic virus 2 (ABB04855); CmCV, *Cucumis melo* cryptic virus (QBC66121).

Sequence analysis of *P. vexans* partitivirus 1 (PvPV1) revealed two independent segments (dsRNA1 and dsRNA2), each with a single open reading frame (ORF) (**Figure 12A**). The full-length dsRNA1 was 1662 nt, containing an ORF encoding a 518-aa protein of RDRP with a molecular weight of 59.8 kDa. The 5’ and 3’ untranslated regions (UTRs) of dsRNA1 were 45 nt and 60 nt, respectively. BLASTp alignment of the ORF encoded by dsRNA1 indicated that the ORF encoded by dsRNA1 was most similar to RdRp in the family Partitiviridae, including diatom colony-associated dsRNA virus 14 (DcaRV14; identity, 48.23%; query cover, 92%; E-value, 4e-148), pepper cryptic virus 1 (PcV1; identity, 36.92%; query cover, 86%; E-value, 6e-93), *Raphanus sativus* cryptic virus 2 (RsCV2; identity, 37.50%; query cover, 86%; E-value, 1e-89), and *Cucumis melo* cryptic virus (CmCV; identity, 36.12%; query cover, 84%; E-value, 37 1e-88). The results of a conservative structure database (CDD) search and multiple alignment (**Figure 12B**) revealed 6 (III to VIII) conserved domains. The sequence of dsRNA1 was deposited in GenBank under accession number MT711194.

The full-length dsRNA2 was 1628 nts and had a C+G content of 42.86%, and the base numbers of the 5’ and 3’-UTRs were 168 nt and 263 nt, respectively. In addition, it contained an ORF (169 nt-1366 nt) encoding a hypothetical protein with a molecular weight of 44.5 kDa. BLASTp alignment of the ORF encoded by dsRNA2 indicated that the ORF of PvPV1 was most closely related to DcaRV14 (identity, 24.10%; query cover, 92%; E-value, 2e-10) in Deltapartitivirus. The sequence of dsRNA2 was deposited in GenBank under accession number MT711195.

**Figure 13:**
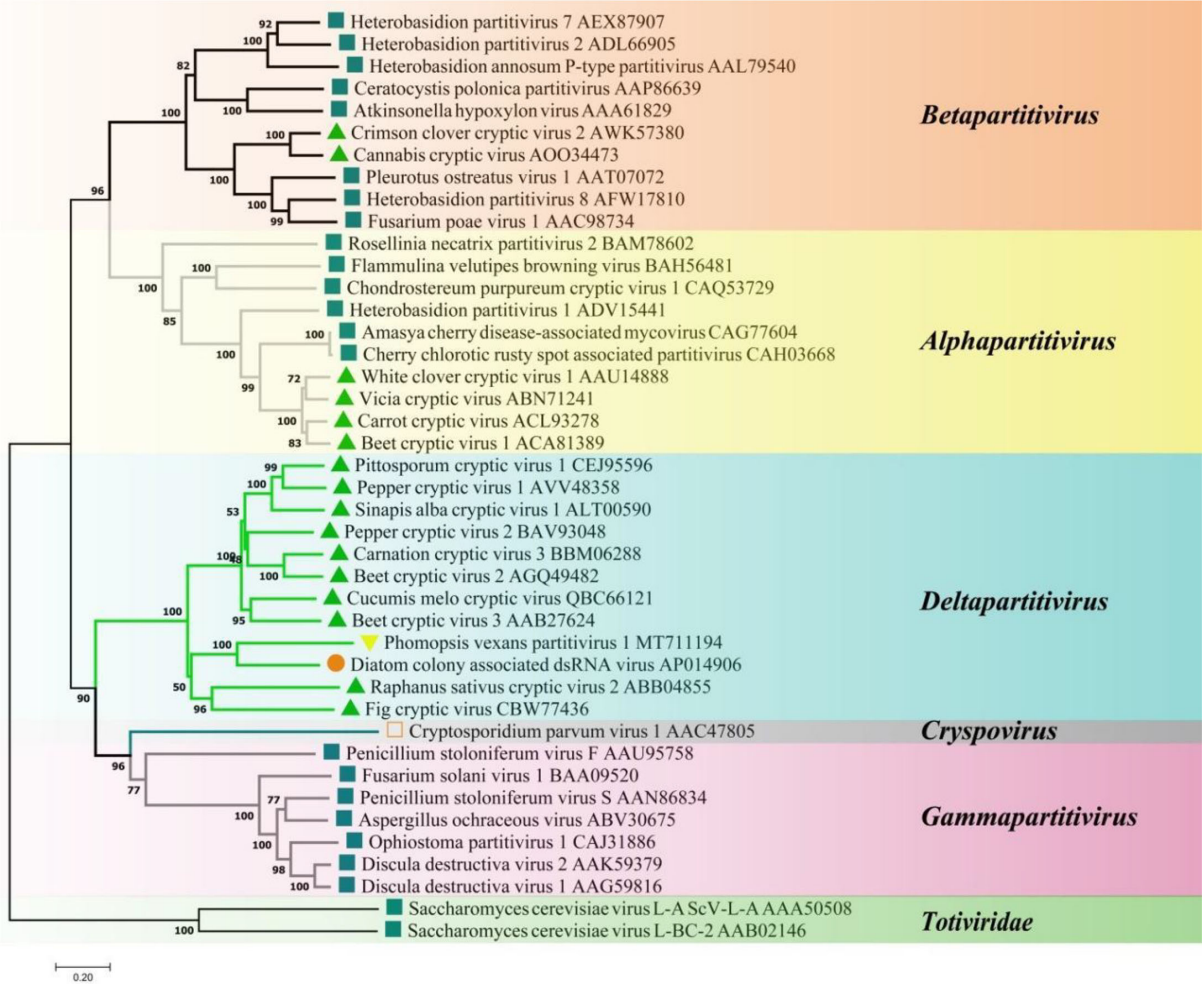
Phylogenetic analysis of PvPV1. Phylogenetic analysis illustrating the evolutionary classification of PvPV1 was conducted using the method described in **Figure 7**. The scale bar represents a genetic distance of 0.2 aa substitutions per site. Yellow triangles indicate the novel mycoviruses PvPV1 identified in the present study. The symbols indicate the viruses isolated from the same group of hosts, such as plants (green triangle), fungi (blue square), and protozoa (orange square). The names and database accession numbers of other related viruses analyzed are indicated in the tree.

A phylogenetic tree based on PvPV1 encoding RdRp was constructed by the neighbor-joining algorithm using MEGA7, with 1000 bootstrap replications (**Figure 13**). The blue square represents the identified virus in the fungus, and the green triangle represents the identified virus in the plant. Phylogenetic analysis showed that PvPV1 and deltapartitivirus in the family Partitiviridae were clustered together. In addition, it has been reported that the hosts of deltapartitivirus and cryspovirus viruses are plants and protozoa (Nibert et al., 2009; Vainio et al., 2018). This is the first report to describe deltapartitivirus virus in fungi.

#### 3.4.5 Structural analysis of the PvVV L1 genome

**Figure 14:**
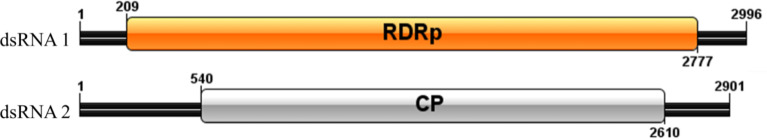
Diagrammatic representation of the genome organization of PvVV L1.

**Figure 15:**
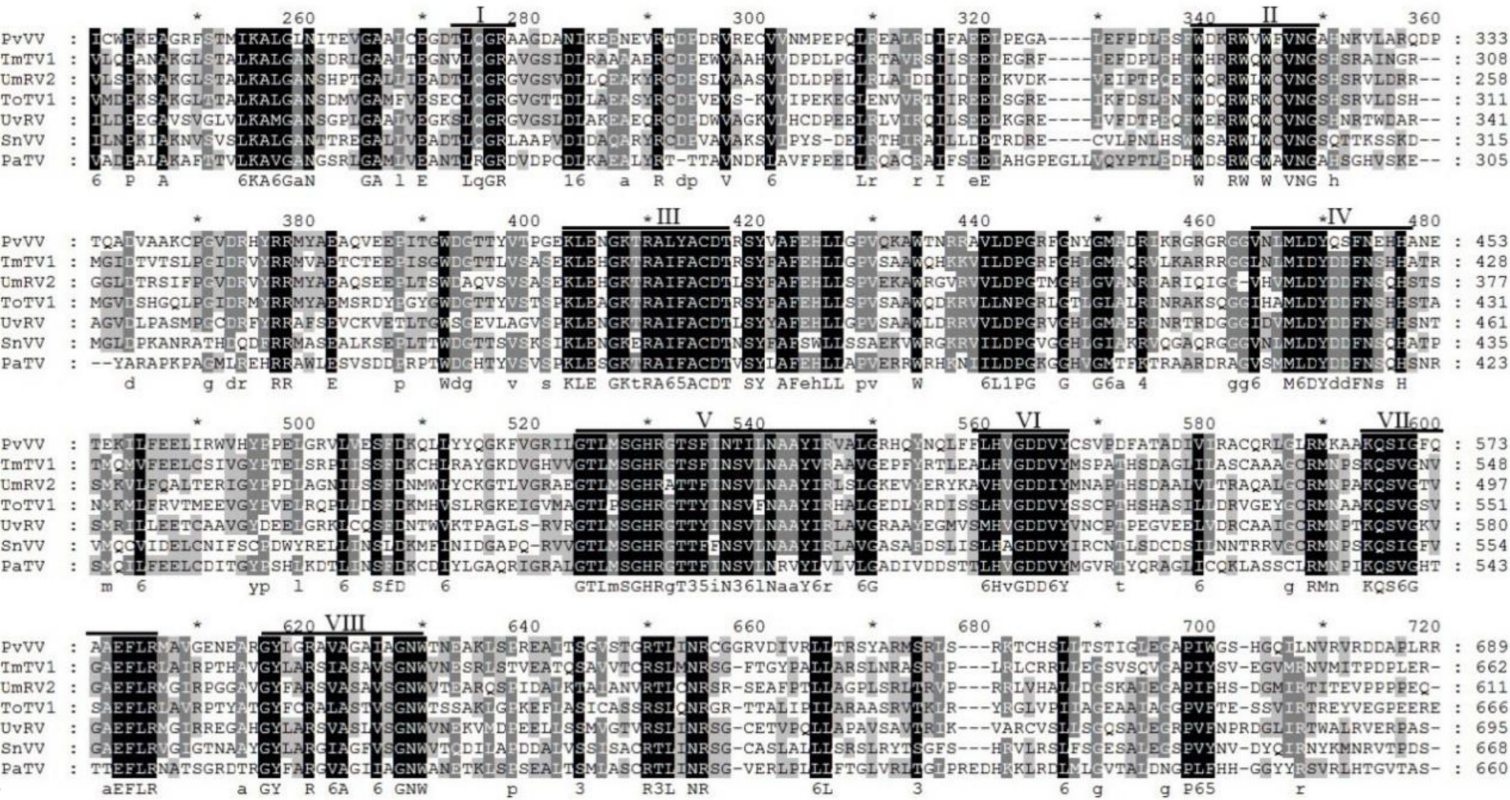
Multiple sequence alignment of the aa sequences of the RdRp domains of PvVVL1 and other similar victoriviruses using the ClustalX program and highlighted using the GeneDoc program. The conserved motifs in these RdRps are indicated by Roman numerals I to VIII.

Sequence analysis of *P. vexans* partitivirus 1 (PvVVL1) revealed two independent segments (dsRNA1 and dsRNA2), each with a single open reading frame (ORF) (**Figure 14**). The full-length dsRNA1 was 2996 nts, and the GC content was 56.4%, containing an ORF encoding an 855-aa protein of RDRP with a molecular weight of 95.2 kDa. The 5’ and 3’ untranslated regions (UTRs) of dsRNA1 were 209 nt and 219 nt, respectively. BLASTp alignment indicated that the ORF encoded by dsRNA1 was most similar to RdRp of victorivirus in the family Totiviridae, including lebolus microspores totivirus 1(TmTV1; identity, 45.42%; query cover, 76%; E-value, 0.0, *Umbelopsis ramanniana* virus 2 (UmRV2; identity, 41.72%; query cover, 76%; E-value, 0.0, *Tolypocladium ophioglossoides* totivirus 1 (ToTV1; identity, 41.68%; query cover, 76%; E-value, 0.0, *Ustilaginoidea virens* RNA virus 5(UvRV5; identity, 41.03%; query cover, 76%; E-value, 7e-175, *Sclerotinia nivalis* victorivirus 1 (SnVV1; identity, 37.95%; query cover, 76%; E-value, 6e-158, *Penicillium aurantiogriseum* totivirus 1 (PaTV1; identity, 38.28%; query cover, 76%; e-value, 5e-157. The conservative structure database (CDD) was searched, and multiple aa comparisons were carried out. The results showed that there were 8 (I to VIII) conserved domains of the monotypic Viridae family (**Figure 15**).

The full-length dsRNA1 was 2901 nts, and the GC content was 59.6%, containing an ORF encoding a 689 aa protein of coat protein (CP) with a molecular weight of 73.5 kDa. The 5’ and 3’ untranslated regions (UTRs) of dsRNA1 were 540 nt and 291 nt, respectively. BLASTp alignment indicated that the ORF encoded by dsRNA2 was most similar to CP in the family Totiviridae, including *Thelebolus microsporus* totivirus 1(TmTV1, *Tolypocladium ophioglossoides* totivirus 1(ToTV1, *Fusarium asiaticum* victorivirus 1 (FaVV1, *Sclerotinia nivalis* victorivirus 1(SnVV1, *Sphaeropsis sapinea* RNA virus 1(SsRV1.

**Figure 16:**
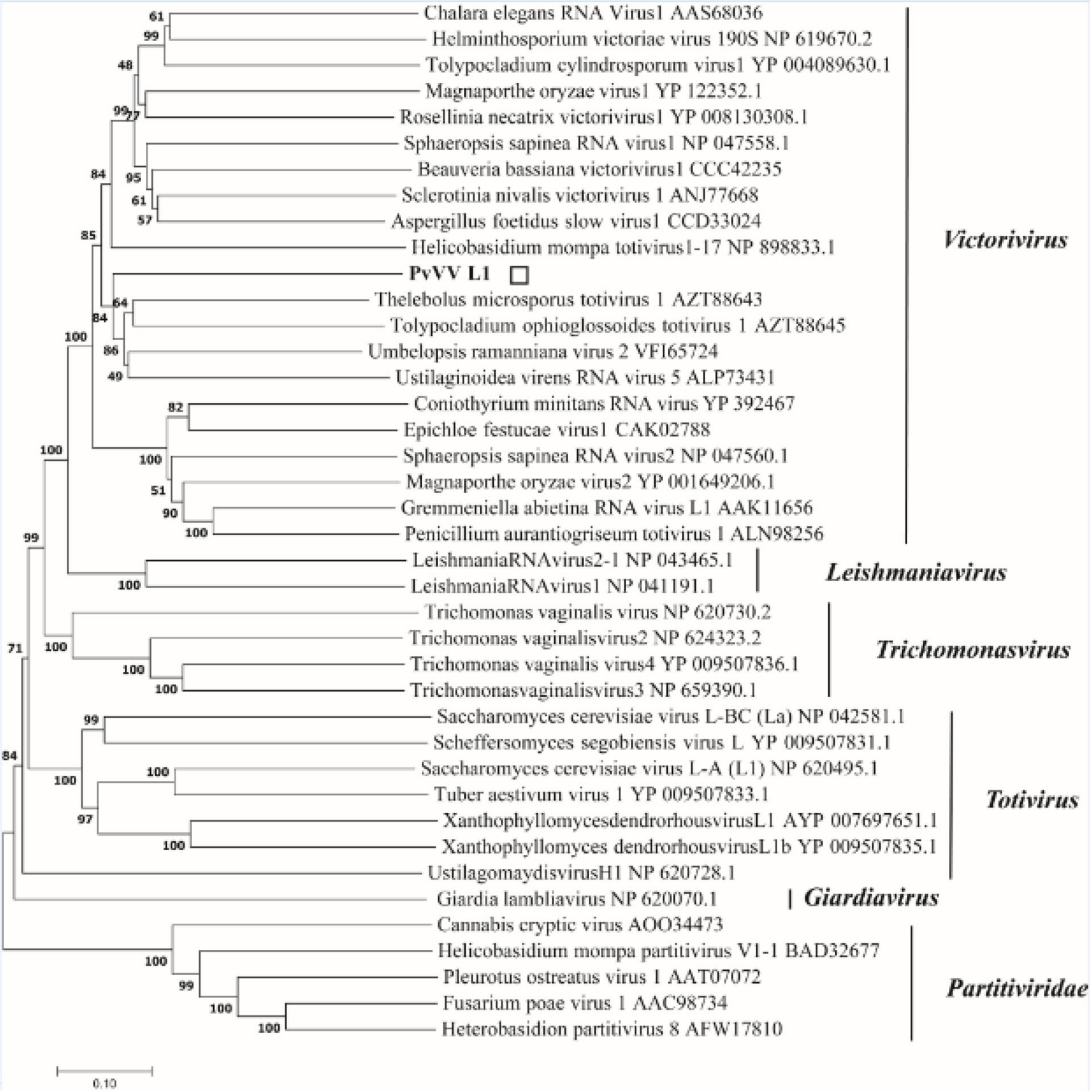
Phylogenetic analysis of PvVVL1. Phylogenetic analysis illustrating the evolutionary classification of PvVVL1 was conducted using the method described in **Figure 7**. The scale bar represents a genetic distance of 0.1 aa substitutions per site. The white square indicates the novel mycovirus PvVVL1 in the present study. The names and database accession numbers of other related viruses analyzed are indicated in the tree.

**Figure 17:**
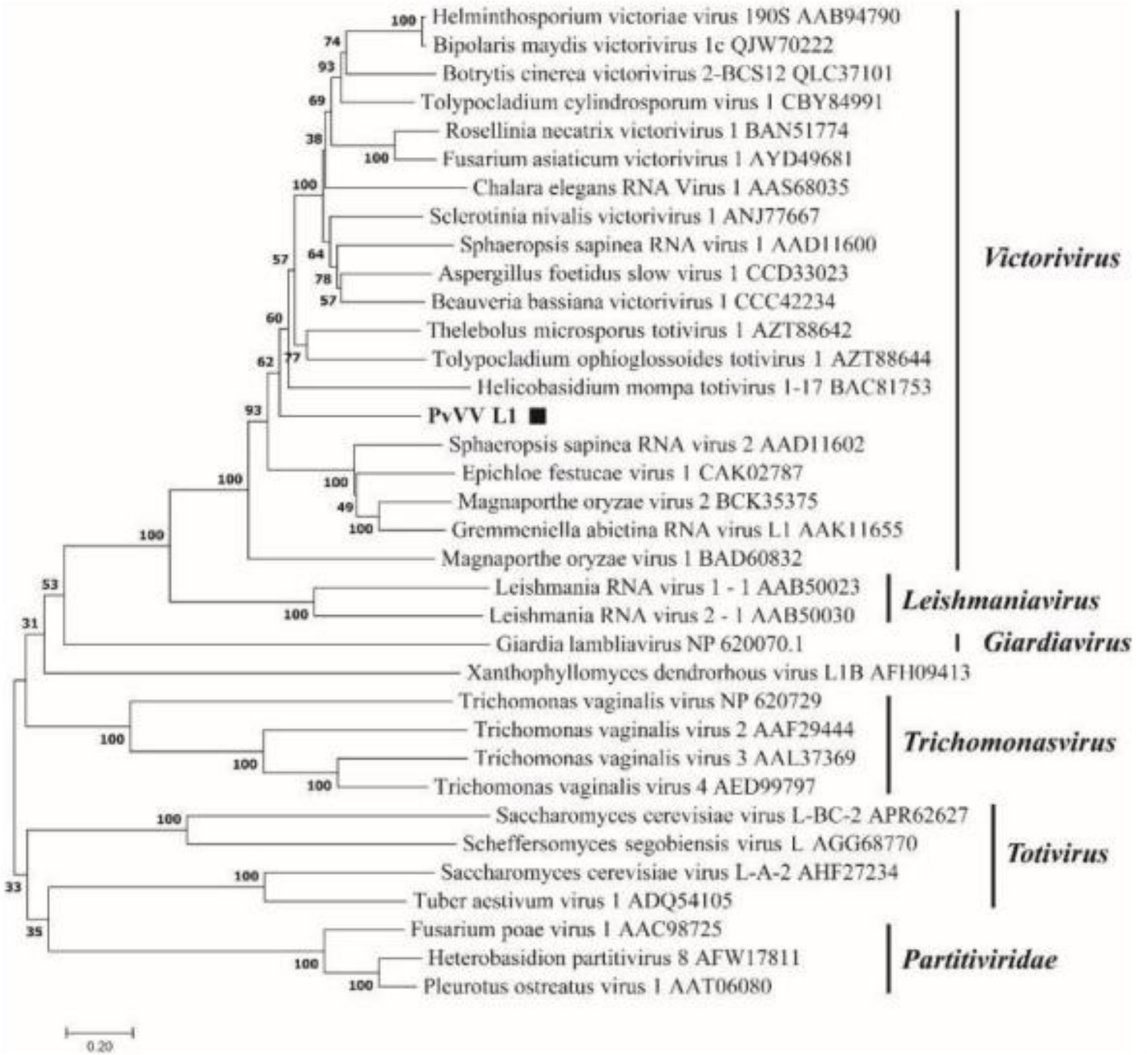
Phylogenetic analysis of PvVVL1. The phylogenetic tree was constructed based on the viral CP aa sequences using the neighbor-joining (NJ) method in MEGA 7 and the Poisson model. The scale bar represents a genetic distance of 0.2 aa substitutions per site. Black squares indicate the novel mycoviruses PvVVL1 identified in the present study.

A phylogenetic tree based on PvVVL1 encoding RdRp and CP aa was constructed by the neighbor-joining algorithm using MEGA7, with 1000 bootstrap replications (**Figures 16**, **17**). The results showed that PvVVL1 and victorivirus of the family Totiviridae were clustered into the same group. Combined with the results of multiple aa alignment of RdRp, PvVVL1 was found to belong to the victoriviruses based on sequence similarity and phylogenetic analysis.

## 4 Discussion

The presence of mycoviruses was detected in all 58 P*. vexans* isolates studied, indicating abundant mycovirus resources. This study provides a potential basis for the biological control of *P. vexans* and increases understanding of the diversity of mycoviruses in *P. vexans*. In this study, we characterized five novel mycoviruses (PvFV1, PvOLV1, PvEV2, PvPV1 and PvVVL1) from *P. vexans* strains and identified diverse mycoviruses using high-throughput transcriptome sequencing. The mycoviruses discovered in our analysis were nearly full length. These detected mycoviruses might belong to the families Botourmiaviridae, Fusariviridae, Endornaviridae, Partitiviridae and Totiviridae. We conducted RT−PCR, genomic PCR and Sanger sequencing analyses using specific primers based on the obtained fragments to confirm the origins of our viral sequences. Positive amplicons were produced from only RNAs extracted from the *P. vexans* strain by RT−PCR but not by genomic PCR, confirming that the viral sequences represented nonintegrated RNA virus sequences. To the best of our knowledge, this is the first report of a comprehensive analysis of viral diversity in *P. vexans* strains. Thus, our results represent a step forward in exploring *P. vexans* mycoviruses and provide insight into screening the potential of *P. vexans* controls and understanding the mechanism of *P. vexans* and mycoviruses coexisting with each other. To our knowledge, mycoviruses reported in *P. vexans* include only Phomopsis vexans RNA virus 1 (PvRV1) (Zhang et al., 2015) and Phomopsis vexans partitivirus 1 (PvPV1) (Zhang et al.). There have been no reports of hypovirulence caused by mycoviruses in the pathogenic fungus *P. vexans*. As long as different fungal taxa and strains are screened for the purpose of discovering novel mycoviruses, our insight into the diversity of mycoviruses will by all means largely increase. In this context, this study provides a rich resource of mycoviruses, which may contain a large number of mycoviruses with biocontrol potential. In follow-up research, we will try to obtain virus-free strains using other methods, such as protoplast regeneration technology, to determine the impact of the four Botourmiaviruses investigated herein on host pathogenicity and the interactions among them.

In addition, as an increasing number of atypical mycoviruses were discovered, they provided a reference for the evolution of mycoviruses. There are two hypotheses regarding the origin of mycoviruses (Pearson et al., 2010): the first is that mycoviruses evolve with host fungi; the second is that mycoviruses may be isolated from the natural host plants of pathogenic fungi. Regarding PvPV1, the available information is limited, and it shows the highest homology to diatom colony-associated dsRNA virus14 deriving from marine organisms. PvPV1 is the first deltapartitivirus reported in fungi. Although PvPV1-specific primers were used to detect the virus in eggplant leaves collected in other fields, it is unfortunate that PvPV1 infection of eggplant plants was not detected. It is worth noting that the Partitiviridae genome consists of two separate dsRNA segments, which encode CP and RdRp, and the virion protein has a molecular weight of 37-77 kDa. The genome of Totiviridae is usually a single linear molecule with a size of 4.6-7.0 kbp encompassing two open reading frames. The 5’-terminal ORF encodes the virus capsid protein, the 3’-terminal ORF encodes a dependent RNA polymerase, and the virion protein has a molecular weight of 70-100 kDa. Expression of the downstream open reading frame is the basis of the Totiviridae classification, and the Totiviridae family has a specific translation mechanism. Victorivirus has a ribosomal insertion site (IRES) in the 5’ noncoding region for CP translation, while the downstream RdRp translates two independent viral proteins through a cis-trans translation mechanism that enables a stop or restart mediated by “AUGA” or “UAAUG”. Totiviridae forms a fusion protein, and viral translation is mediated by the frameshift structure of “- 1 frameshift”. Although atypical victorivirus genomic structures have been reported previously, such as a 98-nt interval between two ORFs in PvRV1 (Zhang et al., 2015) and a 2-nt interval between two ORFs of Magnaporthe oryzae virus 1 in Magnaporthe grisea (Yokoi et al., 2007). It is obvious that there are great differences in the viral genome structure and translation mechanisms between Totiviridae and Partitiviridae. At present, viruses are mainly classified according to their RdRp aa sequences. Through the phylogenetic analysis of PvVVL1, it was found that it was clustered with victoriviruses in the family Totiviridae. Multiple aa alignment analysis showed that the closely related viruses (TmTV1, UmRV2, ToTV1, UvRV5, SnVV1, PaTV1) had eight conserved motifs from the family Totiviridae. The structure of the PvVVL1 genome is composed of two separate dsRNA segments, which is similar to Partitiviridae, indicating a certain evolutionary relationship between the two families of viruses. Ghabrial believes that partitiviruses and totiviruses may share a common ancestor (Ghabrial, 1998). The RdRp of NrVL1 found in N. radicicola has high homology with Partitviridae (Ahn and Lee, 2001), but the genomic structure of the virus is a nondouble segment, and the genome size (6 kbp) is larger than that of Partitviridae, which seems to confirm the view of Ghabrial. At first, the virus may have only RdRp to complete replication in the host, transmission of the virus depends on the transfer of mRNA, and the transmission efficiency of the virus is not high. The virus gradually sequesters the host protein, which is used to encompass and spread RdRp in the form of a virion, forming a multisegmental virus. In recent years, viruses with atypical genomic structures have been reported in fungi. For example, Chiba reported that Narnavirus virus Aspergillus lentulus narnavirus 1 (AleNV1) with a two-segment genome was identified for the first time from Aspergillus (A. fumigatus) using segmented and primer-ligated double stranded RNA sequencing (FLDS) techniques (Chiba et al., 2021). The RdRp of RNA viruses usually encodes an open reading frame, and some viruses of Narnaviridae are often found to lack catalytic domains C and D of RdRp. Chiba found multisegmental Narnavirus viruses, Aspergillus fumigatus narnavirus 2 (AfuNV2) and Magnaporthe oryzae narnavirus 1 (MoNV1), in two different host fungi, Aspergillus and Pyricularia. The missing domains C and D were located at the N-terminus of another RNA segment of the virus. Using SWISS-MODEL software to model the homology of two ORFs, it was found that a functional enzyme could be formed between the two segments, which indicated that the two RNA segments might come from the common segment. In addition, Lin (Lin et al., 2020) and Suvi (Suvi et al., 2020) also described multisegment mycoviruses with mitotic RdRp sequences, indicating that viruses with atypical genomic structures are widely distributed in different host fungi and different countries. The study of multisegment viruses will provide materials for the evolutionary origin of the viruses.

Zhang showed that a dsRNA fragment with a size of approximately 3 kbp and PvRV1 always existed simultaneously and maintained high genetic stability in virus elimination and culture, suggesting that the two dsRNA chains might be related or interact with each other (Zhang et al., 2015). This phenomenon was also observed in 58 tested strains, which confirmed that this phenomenon was not accidental, and it was found in most *P. vexans* strains. Although the electrophoresis results showed that dsRNA of 3 kbp in size and dsRNA of 5 kbp in size did not appear simultaneously, no single dsRNA of 3 kbp in size was observed. According to the electrophoresis results, two dsRNA segments were observed in some strains at the same position, while only one electrophoresis band was observed in some strains, which might be related to the virus type or electrophoresis environment. Therefore, it is speculated that the dsRNA segment at the 3 kbp position might be the defective RNA or satellite RNA segment of a virus. In the long-term experimental process, it was found that the mycoviruses in *P. vexans* could be stably transmitted to progeny through hyphae and spores with high genetic stability. No virus-free wild-type strains were found in the available studies on mycoviruses that infect *P. vexans*, although only 58 strains were isolated and screened, and the probability of carrying the mycoviruses was 100%. These results indicated that the mycoviruses were closely related to the species evolution of *M. aubergine*, and virus transmission occurred frequently among different strains. It was found that eggplant Browning streak was highly genetic conserved (Bhardwaj et al., 2014) and underwent long-distance transmission *via* seeds. Mycoviruses can be transmitted horizontally in homologous strains by mycelial fusion. Some specific mycoviruses can reduce heterologous incompatibility responses and facilitate virus transmission (Wu et al., 2017). There are microbes in nature that can facilitate viral transfer, and CMV has confirmed the existence of transboundary transmission (Andika et al., 2017). Therefore, it is speculated that the mycoviruses carried by the laboratory isolates of *P. vexans* may have a common ancestor. By infecting local *P. vexans*, the virus can be transmitted over a long distance along with the seed circulation of *P. vexans*. After the onset of disease, the virus is transferred to the local population through intermycelial fusion. However, not all viruses can be transmitted by horizontal transfer. Different hosts may have different antiviral mechanisms, and there is also antagonism between viruses. Viruses carried by the host itself will reject the invasion of alien viruses, so viruses need to evolve to complete invasion. Concurrently, there are also types of mutualism between different viruses. Zhang (Zhang et al., 2016) found that through infectious cloning and viral particle transfection, a virus-free ssRNA virus, Yado-Karivirus1 (YkV1), changes its replication pattern by hijacking the viral capsid protein Yado-Nushivirus1 (YnV1). In addition, YkV1 enhances the accumulation of YnV1 in the host. This virus changes its replication mode to suit its own survival to achieve long-term development. The transmissibility of different viruses and the possible different interactions between them also partly explain the diversity of viral RNA bands in different strains of *P. vexans*.

## Data availability statement

The datasets presented in this study can be found in online repositories. The names of the repository/repositories and accession number(s) can be found in the article/**Supplementary Material**.

## Author contributions

HZ, FL,QZ and JZ conceived and designed the experiments. CZ, FX and XZ performed the experiments.FL,HZ and RZ performed the literature search and analyzed the data. FX drafted and revised the manuscript. XZ and CZ contributed equally to writing the manuscript. All authors contributed to the article and approved the submitted version.

## Funding

This work was supported by the China Agriculture Research System of MOF and MARA (CARS-24-A-15), Hunan Provincial Natural Science Foundation of China (2021JJ30 356), Hunan Provincial Science and Technology Innovation Platform Construction Fund (20K071) and Innovation and Entrepreneurship Training Program for College Students (XCX2021049).

## Conflict of interest

The authors declare that the research was conducted in the absence of any commercial or financial relationships that could be construed as a potential conflict of interest.

## Publisher’s note

All claims expressed in this article are solely those of the authors and do not necessarily represent those of their affiliated organizations, or those of the publisher, the editors and the reviewers. Any product that may be evaluated in this article, or claim that may be made by its manufacturer, is not guaranteed or endorsed by the publisher.
