# Supplementary material for: Discovery and exploration of widespread infection of mycoviruses in Phomopsis vexans, the causal agent of phomopsis blight of eggplant in China
Source: Front Plant Sci. 2022 Nov 10;13:996862. doi: 10.3389/fpls.2022.996862 (PMC9685175; doi:10.3389/fpls.2022.996862)
Supplement: Supplementary file 1 [file DataSheet_1.docx]

Supplementary Material


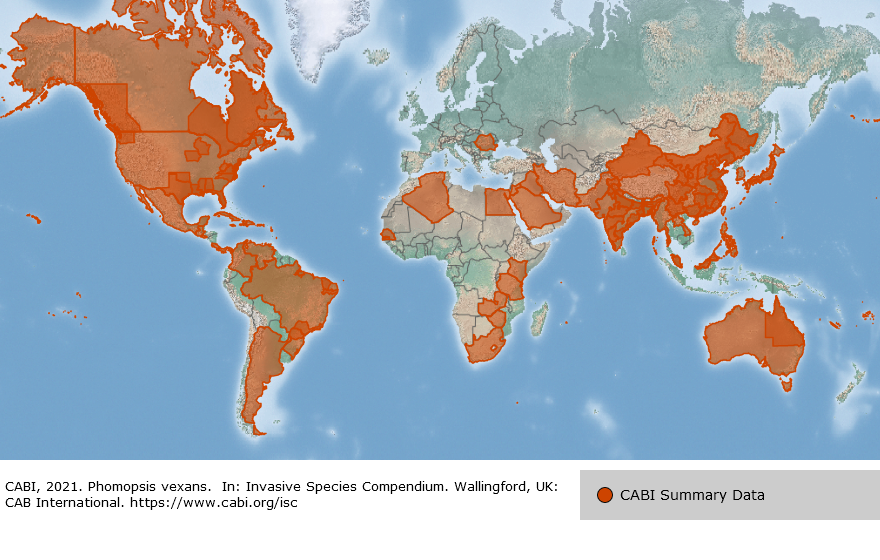


**Supplementary Figure 1.** Global distribution map of Eggplant brown streak.In:Invasive Species Compendium.Wallingford,UK:CAB International.https://www.cabi.org/isc

**Supplementary Table 1.** Source location of the *P. vexans*

| number | Isolation of sample | Sampling time | Sampling position |
| --- | --- | --- | --- |
| PV_1 | leaf | 2017 | Jianggan District,Hangzhou,Zhejiang province |
| PV_2 | leaf | 2017 | Xiaoshan District,Hangzhou,Zhejiang province |
| PV_3 | leaf | 2017 | Qingyuan County,Lishui, Zhejiang province |
| PV_4 | leaf | 2017 | Gaoqiao Town,Changsha , Hunan province |
| PV_5 | leaf | 2017 | Hanshou County, Changde, Hunan province |
| PV_6 | leaf | 2017 | Dongguan,Guangdong province |
| PV_7 | leaf | 2017 | Furong District,Changsha , Hunan province |
| PV_8 | leaf | 2017 | Furong District,Changsha , Hunan province |
| PV_9 | leaf | 2017 | Furong District,Changsha , Hunan province |
| PV_10 | leaf | 2017 | Changsha County,Changsha , Hunan province |
| PV_11 | leaf | 2017 | Wangcheng District,Changsha , Hunan province |
| PV_12 | leaf | 2017 | Da’an Township,Suining,Sichuan province |
| PV_13 | leaf | 2017 | Yuyao,Hangzhou,Zhejiang province |
| PV_14 | leaf | 2017 | Qingyuan,Guangdong province |
| PV_15 | leaf | 2017 | Guangzhou,Guangdong province |
| PV_16 | leaf | 2017 | Yongtai County,Fuzhou,Fujian province |
| PV_17 | fruit | 2017 | Yongtai County,Fuzhou,Fujian province |
| PV_18 | leaf | 2018 | Lion Rock,Wuhan,Hubei province |
| PV_19 | leaf | 2018 | Zhanjiang,Guangdong province |
| PV_20 | leaf | 2018 | Mianyang,Sichuan province |
| PV_21 | leaf | 2018 | Mianyang,Sichuan province |
| PV_22 | leaf | 2018 | Jiangyang District,Luzhou,Sichuan province |
| PV_23 | leaf | 2018 | Jianghua County, Yongzhou, Hunan province |
| PV_24 | leaf | 2018 | Jianghua County, Yongzhou, Hunan province |
| PV_25 | leaf | 2018 | Jieyang,Guangdong province |
| PV_26 | leaf | 2018 | Changsha County,Changsha , Hunan province |
| PV_27 | leaf | 2018 | Da’an Township,Suining,Sichuan province |
| PV_28 | leaf | 2018 | Cangshan,Fuzhou,Fujian province |
| PV_29 | leaf | 2018 | Budang,Fuzhou,Fujian province |
| PV_30 | leaf | 2018 | Jin’an,Fuzhou,Fujian province |
| PV_31 | leaf | 2018 | Ganyao Town,Jiaxing,Zhejiang province |
| PV_32 | leaf | 2018 | Hanshou County, Changde, Hunan province |
| PV_33 | leaf | 2018 | Qianshan County, Anqing, Anhui province |
| PV_34 | leaf | 2018 | Shijiawan,Ningxiang, Hunan province |
| PV_35 | leaf | 2018 | Xundong District,Huzhou,Zhejiang province |
| PV_36 | leaf | 2018 | Leiyang,Hengyang,Hunan province |
| PV_37 | leaf | 2018 | Shimen County, Changde, Hunan province |
| PV_38 | leaf | 2018 | Wenjiang District,Chengdu,Sichuan province |
| PV_39 | leaf | 2018 | Taoyuan County, Changde, Hunan province |
| PV_40 | fruit | 2018 | Linli County, Changde, Hunan province |
| PV_41 | leaf | 2019 | Chaling County, Zhuzhou, Hunan province |
| PV_42 | fruit | 2019 | An’xiang County, Changde, Hunan province |
| PV_43 | leaf | 2019 | Qianshan County, Anqing, Anhui province |
| PV_44 | leaf | 2019 | Qianshan County, Anqing, Anhui province |
| PV_45 | leaf | 2019 | Pingjiang County,Yueyang, Hunan province |
| PV_46 | leaf | 2019 | Fuzhou,Fujian province |
| PV_47 | leaf | 2019 | Fuzhou,Fujian province |
| PV_48 | leaf | 2019 | Dingcheng District, Changde, Hunan province |
| PV_49 | leaf | 2019 | Nan County,Yiyang, Hunan province |
| PV_50 | leaf | 2019 | Shangrao,Jiangxi province |
| PV_51 | leaf | 2019 | Xiangtan, Hunan province |
| PV_52 | leaf | 2019 | Shaoyang, Hunan province |
| PV_53 | leaf | 2019 | Xinning County, Shaoyang, Hunan province |
| PV_54 | fruit | 2019 | Xinning County, Shaoyang, Hunan province |
| PV_55 | fruit | 2019 | Yuping,Jiangxi province |
| PV_56 | leaf | 2019 | Jianghua County, Yongzhou, Hunan province |
| PV_57 | leaf | 2019 | Shuangfeng County, Loudi, Hunan province |
| PV_58 | fruit | 2019 | Lizhou District,Guangyuan,Sichuan province |

**Supplementary Table 2.** Primer and adapter used in this chapter

| Primer | sequence | purpose |
| --- | --- | --- |
| dN6 | 5’-CGATCGATCATGATGCAATGCNNNNNN-3’ | Library construction |
| Specific | 5’-CGATCGATCATGATGCAATGC-3’ | Library construction |
| RACE | 5’-(PO4)TCTCTTCGTGGGCTCTTGCG (NH2)-3’ | obtain the terminal sequences |
| RACE3RT | 5’-CGCAAGAGCCCACGAAGAGA-3’ | obtain the terminal sequences |
| adapter | 5’-(PO4)GCATTGCATCATGATCGATCGAATTCTTTAG TGAGGGTTAATTGCC (NH2)-3’ | obtain the terminal sequences |
| oligoREV | 5’-GGCAATTAACCCTCACTAAAG-3’ | obtain the terminal sequences |
| ligation | 5’-TCACTAAAGAATTCGATCGATC-3’ | obtain the terminal sequences |
